# Localized periodontitis severity associated with carotid intima-media thickness in young adults: CHIEF atherosclerosis study

**DOI:** 10.1038/s41598-023-37840-4

**Published:** 2023-06-29

**Authors:** Kun-Zhe Tsai, Wei-Chun Huang, Yun-Chen Chang, Younghoon Kwon, Xuemei Sui, Carl J. Lavie, Gen-Min Lin

**Affiliations:** 1 Department of Internal Medicine, Hualien Armed Forces General Hospital, No. 100, Jinfeng St., Hualien City, 970 Taiwan; 2 Department of Stomatology of Periodontology, Mackay Memorial Hospital, Taipei, Taiwan; 3 Department of Dentistry, Tri-Service General Hospital and National Defense Medical Center, Taipei, Taiwan; 4 Department of Critical Care Medicine, Kaohsiung Veterans General Hospital, Kaohsiung, Taiwan; 5 School of Nursing and Graduate Institute of Nursing, China Medical University, Taichung, 406 Taiwan; 6 Department of Medicine, University of Washington, Seattle, WA USA; 7 Department of Exercise Science, Arnold School of Public Health, University of South Carolina, Columbia, SC USA; 8 John Ochsner Heart and Vascular Institute, Ochsner Clinical School, The University of Queensland School of Medicine, New Orleans, LA USA; 9 Department of Internal Medicine, Tri-Service General Hospital and National Defense Medical Center, Taipei, Taiwan

**Keywords:** Cardiology, Medical research

## Abstract

This study aimed to investigate the association of periodontitis with subclinical atherosclerosis in young adults. In total, 486 non-diabetic military personnel were included in Taiwan. Carotid intima-media thickness (cIMT) was assessed utilizing sonography for subclinical atherosclerosis. Periodontitis severity was defined based on the 2017 US/European consensus. Mean cIMT was compared by analysis of covariance (ANCOVA), and multiple logistic regression model was used to determine the association of periodontitis severity and the highest quintile of cIMT (≥ 0.8 mm) with adjustments for age, sex, metabolic risk factors and leukocyte counts. The mean cIMT increased in those with greater stages (periodontal health (N = 349): 0.65 mm, Stage I (N = 41): 0.72 mm, Stage II (N = 57): 0.74 mm and Stage III: 0.76 mm, respectively, *p* < 0.01). In multiple logistic regression, a dose–response association from Stage I to Stage III periodontitis for cIMT ≥ 0.8 mm was also found [ORs and 95% CIs 1.41 (0.60–3.29), 1.62 (0.79–3.31) and 3.20 (1.42–7.18)]. Leucocyte counts ≥ 7.6 × 10^3^/µL (the highest quintile) was associated with cIMT ≥ 0.8 mm [OR 1.86 (1.11–3.12)], while no association existed for other metabolic risk factors. In conclusion, severe periodontitis and leukocyte counts are independent risk factors of increased cIMT, emphasizing the critical role of inflammation in subclinical atherosclerosis.

## Introduction

Atherosclerosis, the main process in atherosclerotic cardiovascular diseases, is a focal thickening of the arterial intima residing between the endothelial lining and smooth muscle layers of arterial blood vessels in response to an immune response^1^. Periodontitis is associated with disproportionate host inflammatory immune responses that are induced by an imbalance in the microbial ecological environment in oral cavity; this instigates microbial dysbiosis, along with failed resolution of the chronic destructive inflammation^2^. The European Federation of Periodontology (EFP) and the American Academy of Periodontology (AAP) joint workshop in 2012 suggests a possible link between atherosclerotic disease and periodontitis^3^. Two mechanisms are proposed to explain such association. First, the subgingival microbiota and their products may directly access to the circulation and provide a persistent gram-negative bacterial challenge to the host^4^. Low level bacteremia may initiate host responses that alter coagulability, endothelial and vessel wall integrity, and platelet function, thereby resulting in subsequent atherogenic changes and thromboembolic event in patients with periodontitis^5–7^. Second, periodontitis can indirectly alter systemic homeostasis through the release of endotoxins as well as inflammatory cytokines, and lead to endothelial dysfunction^8^. In addition, in epidemiology, both atherosclerosis and periodontitis may be modified by similar risk factors, e.g., age, elements of tobacco and metabolic disorders^9,10^. However, the findings for the association between periodontitis and atherosclerosis markers, i.e., carotid intima-media thickness (cIMT) were inconsistent among middle- and old-aged individuals in previous reports^11,12^. The conflicting results might be due in part to many risk factors prevalently presented in the process of atherosclerosis and periodontitis development in older individuals, and the association might be over-adjusted if these risk factors were all included as confounders. It is critical to carry out a study in a population with low vascular risk factors exposure, e.g., military subjects who are healthier and have better physical activity than the general population, and are unlikely to have atherosclerosis, to verify the association. Therefore, this study aimed to investigate the association between periodontitis severity and cIMT, a marker of subclinical atherosclerosis, in a population of military young adults in Taiwan.

## Methods

### Study population

This study was a cross-sectional study including participants without taking any antihypertensive medications from the cardiorespiratory fitness and health in eastern armed forces (CHIEF) atherosclerosis study in Hualien City, Taiwan in 2018–2020, which has been described in detail previously^13–16^. Participants who underwent both an oral examination and a carotid ultrasonography were included. Participants whose age older than 40 years and total leukocyte counts greater than 11.0 × 10^3^/µL were excluded to avoid a presence of unrecognized infectious diseases in the body. The study protocol was approved by the Ethics Committee of the Mennonite Christian Hospital (No. 16-05-008) in the Hualien City of Taiwan and the study was conducted in accordance with the Helsinki Declaration of 1975, as revised in 2013. Participants were informed the nature of the study, and all gave written informed consent to provide the ability to opt out without consequence.

### Assessment of anthropometric and hemodynamic variables

Waist circumference, body height and body weight were assessed in a standing position. The body mass index (BMI) was defined as body weight in kilograms divided by square of body height in meters. Abdominal obesity was defined as waist circumference ≥ 90 cm for men and ≥ 80 cm for women^17^. Blood samples were collected in the morning after an overnight fast for total cholesterol, low density lipoprotein (LDL-C), high density lipoprotein (HDL-C), triglycerides, fasting glucose (FPG), serum uric acid and leucocyte counts. Dyslipidemia was defined as total cholesterol ≥ 200 mg/dL, LDL-C > 100 mg/dL, or triglycerides ≥ 150 mg/dL in both men and women. HDL-C < 40 mg/dL in men and HDL-C < 50 mg/dL in women were also defined as dyslipidemia^17^. Hyperuricemia was defined as serum uric acid ≥ 7.0 mg/dL in men and ≥ 6.0 mg/dL in women^18^. Systemic inflammation was defined as elevated leucocyte counts ≥ 7.6 10^3^/µL^19^. Measurement of systolic blood pressure (SBP)/diastolic blood pressure (DBP) at rest over right upper arm was performed once, using an automatic device (FT201 Parama-Tech Co., Ltd, Fukuoka, Japan). Hypertension was diagnosed as SBP ≥ 130 mmHg and/or DBP ≥ 85 mmHg^17^. Before the physical examination, participants were asked about their alcohol consumption and tobacco smoking status (active versus former/never)^20,21^, and daily tooth brushing frequency.

### Assessment of periodontitis severity

The 2017 AAP/EFP joint workshop on the classification of periodontal diseases were used for the definition of periodontitis stages^22^. Since the study was aimed at physically fit young adults, the extent of periodontitis was merely restricted to the localized (< 30% of teeth involved)^22^. Stage I periodontitis was defined if either one criterion was fulfilled: (1) interdental clinical attachment loss (CAL) of 1–2 mm at side of greatest loss; (2) radiographic bone loss < 15%; (3) no tooth loss due to periodontitis; (4) maximum probing pocket depth (PD) ≤ 4 mm, or mostly horizontal bone loss. Stage II periodontitis was defined when either one criterion was fulfilled: (1) interdental CAL of 3–4 mm at side of greatest loss; (2) radiographic bone loss of 15–33%; (3) no tooth loss due to periodontitis; (4) maximum PD ≤ 5 mm, or mostly horizontal bone loss. Stage III periodontitis was defined if either one criterion was met: (1) interdental CAL ≥ 5 mm at side of greatest loss; (2) radiographic bone loss extending to mid-third of root and beyond; (3) with tooth loss because of periodontitis; (4) in addition to Stage II periodontitis severity further with PD ≥ 6 mm, or vertical bone loss ≥ 3 mm, or class II/III furcation involvement, or moderate ridge defect. No cases of Stage IV periodontitis were observed in this study. Full mouth oral examination and radiograph of each participant were performed by the same dentist. All subjects returned to the outpatient department for a re-evaluation and a treatment for the oral pathologies within one month. The inter-observer agreement (kappa coefficient) for verifying the severity of periodontitis was estimated 90.6%^23–26^.

### Assessment of cIMT

All ultrasonographic images were acquired at end diastole (defined as the R wave of an electrocardiogram) by the same certificated technician and verified by a cardiologist. The cIMT diameter was quantitatively measured between the leading edges of the media–adventitia interface and the lumen–intima interface over the far wall of the left carotid bulb, using an ultrasound scanner equipped with a linear *p* robe of 4–8 MHz on the iE33 machine (Philips Medical Systems, Andover, MA, USA). Intima–media interface diameter of each participant was manually traced as a continuous line for a calculation^13^. To verify the cIMT, the measurement procedure was performed repeatedly by the technician, and the coefficient of variation was up to 96.8%^14^. In whole of the carotid sonographic examinations, no carotid plagues were found. The increased cIMT was defined as those over the highest quintile level, corresponding to the diameter ≥ 0.8 mm, which was modestly greater than the upper normal cIMT level (0.75 mm) in the general population in prior studies^27^.

### Statistical analysis

Periodontitis severity was classified as health, Stage I, Stage II and Stage III. Group differences among the four groups were evaluated using the analysis of variance (ANOVA) for continuous variables and chi-square test for categorical variables. Analysis of covariance (ANCOVA) with adjustments for age, sex, BMI, SBP, DBP, tooth brushing frequency, alcohol intake and tobacco smoking was used to examine if a difference in the mean of cIMT existed among the four groups. We utilized univariate and stepwise multiple linear regression models to assess the association of periodontitis severity with cIMT. In model 1, age, sex, SBP, DBP, waist circumference, lipid profiles, FPG, serum uric acid, tooth brushing frequency, alcohol intake and smoking status and leukocyte counts were adjusted. In model 2, BMI was further adjusted. Univariate and multiple logistic regression models were also utilized to estimate the odds ratios (ORs) and 95% confidence intervals (CIs) of periodontitis severity for increased cIMT ≥ 0.8 mm. In Model 1, age, sex, teeth brush frequency, alcohol intake, tobacco smoking, metabolic syndrome risk factors, e.g., dyslipidemia and hypertension, and elevated leukocyte counts within normal limits were adjusted. In model 2, BMI was additionally adjusted. In addition, participants were divided into four groups, based on the presence( +) or absence(−) of elevated leukocyte counts and severer periodontitis (Stage II or Stage III) to investigate the moderation effect of elevated leukocyte counts in the association between severer periodontitis and increased cIMT ≥ 0.8 mm in the univariate and multiple logistic regression models. Since the sample of the male participants in this study were much more than the female participants, sensitivity analyses were performed for men only with regard to their clinical characteristics and the association of periodontitis severity with increased cIMT ≥ 0.8 mm. The statistical analysis was carried out using SPSS software (Version 26.0. Armonk, New York: International Business Machines Corporation), and a value of *p* < 0.05 was considered to be statistically significance.

## Results

### Demographic and clinical characteristics

**Figure 1:**
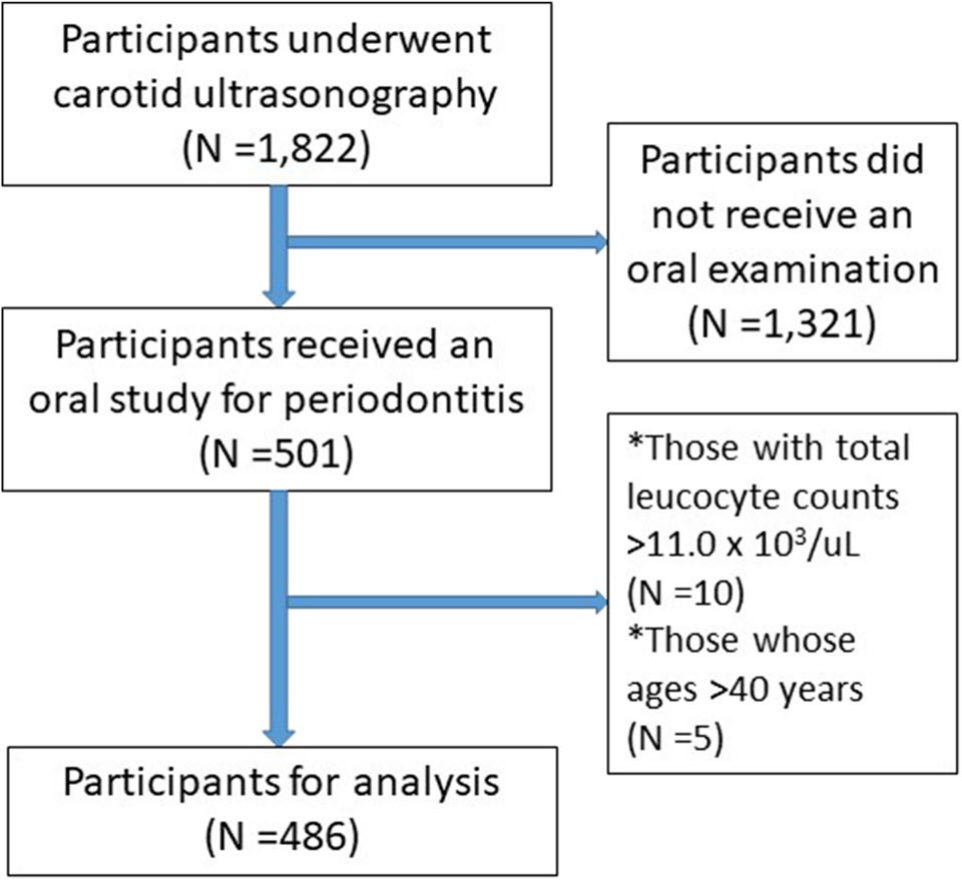
shows the flow diagram for the selection process of the participants in this study.

**Figure 2:**
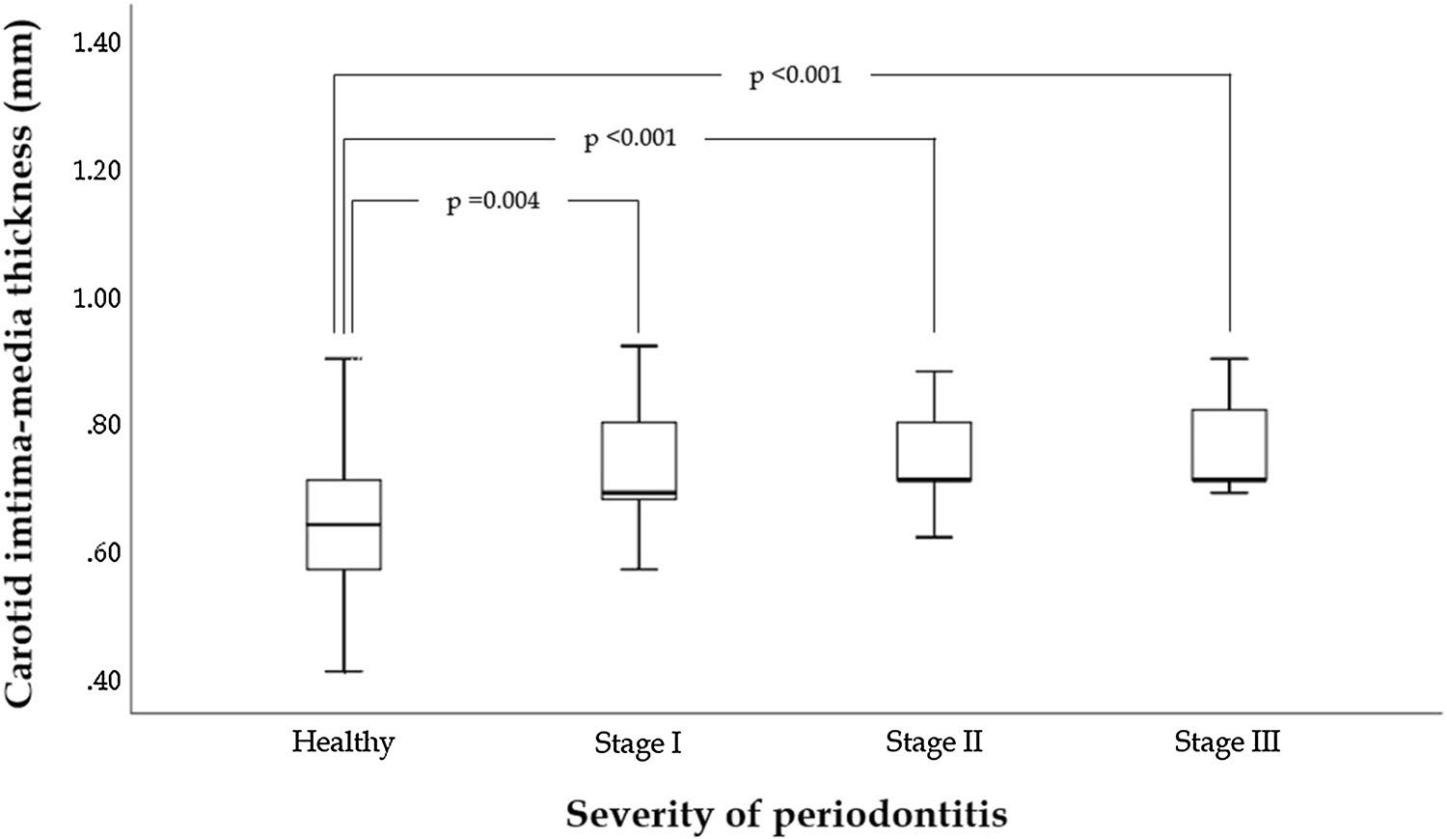
The cIMT was highest in those with Stage III periodontitis (0.76 ± 0.17 mm), followed by those with Stage II periodontitis (0.74 ± 0.05 mm), then those with Stage I periodontitis (0.72 ± 0.08 mm) and lowest in those with periodontal health (0.65 ± 0.12 mm) which was the reference group with adjustments for potential confounders (all *p*-values < 0.05).

Of those who underwent a carotid ultrasonography for cIMT measurement (N = 1,822), participants who received an oral examination were included (N = 501). Those who were older than 40 years of age (N = 5) and those with total leucocyte counts > 11.0 × 10^3^/µL (N = 10) were excluded, leaving a sample of 486 subjects for analysis (Fig. 1). Table 1 shows that the prevalence of periodontitis (any grades) was 28.19% (N = 137), and the others (N = 349) were periodontal health (71.81%). Of those with periodontitis, there were 41 cases (6.62%) classified to Stage I, 57 cases (12.18%) classified to Stage II and 39 cases (8.33%) classified to Stage III. Of the military subjects, about 80% had the behavior of tooth brushing > 1 time per day. There were statistically differences in age, waist circumference, BMI, SBP, DBP, total cholesterol, LDL-C, serum uric acid, FPG and triglycerides. Participants with Stage III periodontitis were found with the highest BMI, waist circumference, SBP, DBP and total cholesterol in the four groups. The results of ANCOVA were shown in Fig. 2. The highest cIMT level was found in those with Stage III periodontitis (0.76 ± 0.17 mm), followed by those with Stage II periodontitis (0.74 ± 0.05 mm), and then those with Stage I periodontitis (0.72 ± 0.08 mm) and lowest in those with periodontal health (0.65 ± 0.12 mm) with adjustments for the confounders (all *p*-values < 0.05). The clinical characteristics of the men are shown in supplemental Table 1, which are similar to that of the whole population of both men and women.

**Table 1:** Clinical characteristics of military young adults receiving oral examination and carotid ultrasonography.

	Periodontal healthy (N = 349)	Periodontitis (N = 137)			*p* value
		Stage I (N = 41)	Stage II (N = 57)	Stage III (N = 39)	
cIMT, mm	0.65 ± 0.12	0.72 ± 0.08	0.74 ± 0.05	0.76 ± 0.17	< 0.001
Age, years	30.65 ± 5.17	36.37 ± 3.84	29.65 ± 5.51	34.18 ± 4.37	< 0.001
Men, %	319 (91.4)	38 (92.7)	56 (98.2)	39 (100.0)	0.08
Alcohol drinking, %	203 (58.2)	23 (56.1)	23 (40.4)	25 (64.1)	0.06
Tobacco smoking, %	231 (66.2)	26 (63.4)	21 (36.8)	20 (51.3)	0.19
Body mass index, kg/m^2^	24.72 ± 2.95	26.23 ± 3.82	26.60 ± 3.96	28.17 ± 3.69	< 0.001
Waist circumference, cm	83.31 ± 7.52	88.12 ± 9.40	86.68 ± 10.57	92.48 ± 9.26	< 0.001
Systolic blood pressure, mmHg	119.32 ± 13.39	119.00 ± 13.02	124.77 ± 18.14	124.87 ± 12.78	0.008
Diastolic blood pressure, mmHg	71.65 ± 10.71	75.08 ± 8.48	75.38 ± 14.64	77.33 ± 10.84	0.002
Blood examinations					
Total cholesterol, mg/dL	170.28 ± 28.93	189.32 ± 28.08	190.58 ± 43.37	196.44 ± 41.13	< 0.001
LDL-C, mg/dL	102.40 ± 25.30	119.95 ± 23.46	114.77 ± 35.79	115.41 ± 32.50	< 0.001
HDL-C, mg/dL	48.95 ± 10.22	48.73 ± 9.13	49.02 ± 9.83	48.41 ± 9.56	0.98
Triglycerides, mg/dL	103.21 ± 66.49	142.56 ± 82.16	153.42 ± 139.74	181.18 ± 163.88	< 0.001
Fasting glucose, mg/dL	92.60 ± 8.08	103.12 ± 12.95	95.14 ± 37.05	104.23 ± 14.97	< 0.001
Uric acid, mg/dL	6.82 ± 1.39	6.85 ± 1.60	6.24 ± 1.26	6.40 ± 1.51	0.01
Leucocyte counts, 10^3^/µL	6.68 ± 1.67	6.74 ± 1.29	6.27 ± 2.07	6.58 ± 1.42	0.36
Tooth brushing frequency					
1 time/day	82 (23.5)	6 (14.6)	7 (12.3)	7 (17.9)	0.25
2 times/day	150 (43.0)	21 (51.2)	26 (45.6)	22 (56.4)	
≥ 3 times/day	117 (33.5)	14 (34.1)	24 (42.1)	10 (25.6)	

Continuous variables are expressed as mean ± SD (standard deviation), and categorical variables as N (%).

*cIMT* carotid intima-media thickness, *LDL-C* low density lipoprotein cholesterol, *HDL-C* high density lipoprotein cholesterol.

### Associations of periodontitis stages and lipid profiles with cIMT levels

The results of the univariate and multiple linear regression analyses for the association of periodontitis severity with cIMT were revealed in Table 2. In the univariate linear analysis, Stage I periodontitis, Stage II periodontitis and Stage III periodontitis, waist circumference, SBP, DBP, triglycerides, total cholesterol, LDL-C, age and BMI were positively and significantly associated with cIMT. In the multiple linear analysis Model 1, Stage I, Stage II and Stage III periodontitis were positively and significantly associated with cIMT levels, and the strength of association increased with greater periodontitis Stage (β = 0.066 (0.025, 0.107), 0.085 (0.050, 0.120) and 0.107 (0.065, 0.149), all *p*-values < 0.01, respectively). Of the metabolic variables, only total cholesterol and LDL-C were associated with cIMT levels ((β = 0.001 (0.000, 0.003) and − 0.001 (− 0.002, 0.000), respectively, both *p*-values < 0.05), while there were no associations for waist circumference, SBP and DBP. The main results were not changed in Model 2.

**Table 2:** Multiple linear regression analysis between periodontitis severity and carotid intima-media thickness in military young adults.

	Crude model				Model 1				Model 2			
	r	β	95% CI	*p* value	r	β	95% CI	*p* value	r	β	95% CI	*p* value
Periodontitis												
Stage I	0.328	0.066	0.028–0.104	0.001	0.374	0.066	0.025–0.107	0.002	0.374	0.066	0.025–0.107	0.002
Stage II		0.089	0.056–0.122	< 0.001		0.085	0.050–0.120	< 0.001		0.085	0.049–0.120	< 0.001
Stage III		0.111	0.071–0.150	< 0.001		0.107	0.065–0.150	< 0.001		0.107	0.065–0.150	< 0.001
*p* value for trend				< 0.001				< 0.001				< 0.001
Waist circumference	0.100	0.001	0.000–0.003	0.02		− 0.001	− 0.002–0.001	0.51		− 0.001	− 0.003–0.002	0.66
Systolic blood pressure	0.090	0.001	0.000–0.002	0.04		0.000	− 0.001–0.001	0.51		0.000	− 0.001–0.001	0.53
Diastolic blood pressure	0.126	0.001	0.000–0.002	0.005		0.001	− 0.001–0.002	0.48		0.000	− 0.001–0.002	0.48
Total cholesterol	0.175	0.001	0.0–0.001	< 0.001		0.001	0.000–0.003	0.008		0.001	0.000–0.003	0.008
LDL-C	0.100	0.000	0.000–0.001	0.02		− 0.001	− 0.002–0.000	0.02		− 0.001	− 0.002–0.000	0.02
HDL-C	0.016	0.000	− 0.001–0.001	0.72		− 0.001	− 0.003–0.001	0.16		− 0.001	− 0.003–0.001	0.17
Triglycerides	0.128	0.000	0.000–0.000	0.005		0.000	0.000–0.000	0.19		0.000	0.000–0.000	0.19
Fasting glucose	0.060	0.000	0.000–0.001	0.18		0.000	− 0.001–0.000	0.30		0.000	− 0.001–0.000	0.30
Uric acid	0.021	0.002	− 0.006–0.010	0.64		0.005	− 0.002–0.013	0.17		0.005	− 0.002–0.013	0.17
Leucocyte counts	0.036	0.003	0.001–0.004	0.43		0.004	0.001–0.005	0.26		0.004	0.001–0.005	0.26
Age	0.097	0.002	0.000–0.004	0.03		0.001	− 0.001–0.003	0.43		0.001	− 0.001–0.003	0.43
Men	0.029	− 0.014	− 0.058–0.030	0.52		− 0.004	− 0.049–0.042	0.87		− 0.004	− 0.050–0.042	0.86
Alcohol intake	0.026	− 0.007	− 0.029–0.016	0.56		− 0.014	− 0.038–0.011	0.27		− 0.014	− 0.039–0.011	0.27
Tobacco smoking	0.040	− 0.010	− 0.034–0.013	0.37		− 0.008	− 0.033–0.017	0.54		− 0.008	− 0.033–0.018	0.55
Tooth brushing frequency	0.066	0.011	− 0.004–0.026	0.14		0.004	− 0.012–0021	0.60		0.004	− 0.012–0.021	0.60
Body mass index	0.114	0.004	0.001–0.007	0.01						0.000	− 0.006–0.007	0.95

Data are presented as standardized β values and 95% confidence intervals (CI).

*LDL-C* low density lipoprotein cholesterol, *HDL-C* high density lipoprotein cholesterol.

### The independent risk factors of increased cIMT

The results of the univariate and multiple logistic regression analyses models of periodontitis severity, systemic inflammation and metabolic syndrome risk factors for increased cIMT are revealed in Table 3. In the univariate logistic regression analysis, participants with Stage III periodontitis, abdominal obesity, hypertension, hyperlipidemia and systemic inflammation were more likely to have cIMT ≥ 0.8 mm. In the multiple logistic regression analysis Model 1, the strength of the association for cIMT ≥ 0.8 mm increased from Stage I, Stage II and Stage III periodontitis [ORs 1.41 (0.60, 3.30), 1.62 (0.79, 3.29), and 3.19 (1.42, 7.14), respectively; *p*-value for trend = 0.001]. Of the other risk factors, systemic inflammation reflected by total leucocyte counts ≥ 7.6 10^3^/µL was the only risk factor associated with cIMT ≥ 0.8 mm [OR 1.86 (1.11, 3.10)]. Notably, there were no associations for the metabolic syndrome risk factors, e.g., abdominal obesity and hypertension. The main results were not changed in Model 2. With regard to the sensitivity analysis for men only (supplemental Table 2), the main results were consistent to that for the whole population of men and women.

**Table 3:** Multiple logistic regression analysis between periodontitis severity and carotid intima-media thickness ≥ 0.8 mm in military young adults.

	Crude model			Model 1			Model 2		
	OR	95% CI	*p* value	OR	95% CI	*p* value	OR	95% CI	*p* value
Periodontitis									
Stage I	1.73	0.82–3.64	0.14	1.41	0.60–3.30	0.42	1.41	0.60–3.29	0.43
Stage II	1.84	0.97–3.50	0.06	1.62	0.79–3.29	0.18	1.62	0.79–3.31	0.18
Stage III	3.65	1.83–7.28	< 0.001	3.19	1.42–7.14	0.005	3.20	1.42–7.18	0.005
*p* value for trend			< 0.001			0.001			0.004
Central obesity (waist circumference ≥ 90 cm for men and ≥ 80 cm for women)	1.82	1.17–2.85	0.008	1.15	0.68–1.95	0.61	1.19	0.59–2.39	0.63
Hypertension (blood pressure ≥ 130/85 mmHg)	1.50	0.96–2.33	0.07	1.34	0.81–2.20	0.25	1.35	0.81–2.23	0.25
Total cholesterol ≥ 200 mg/dL	2.12	1.35–3.43	0.001	1.42	0.77–2.62	0.26	1.42	0.77–2.61	0.26
LDL-C > 100 mg/dL	1.60	1.02–2.49	0.04	1.23	0.71–2.12	0.46	1.23	0.71–2.14	0.45
HDL-C < 40 mg/dL	1.07	0.60–1.89	0.83	1.00	0.53–1.88	0.99	1.00	0.53–1.88	0.99
Triglycerides ≥ 150 mg/dL	1.68	1.03–2.72	0.03	1.03	0.57–1.86	0.92	1.04	0.57–1.89	0.90
Fasting glucose ≥ 100 mg/dL	1.15	0.70–1.90	0.57	0.73	0.40–1.32	0.29	0.73	0.40–1.33	0.29
Hyperuricemia (uric acid ≥ 7.0 mg/dL for men and ≥ 6.0 mg/dL for women)	1.20	0.78–1.86	0.40	1.26	0.79–2.02	0.33	1.26	0.79–2.03	0.33
Leucocyte counts ≥ 7.6 10^3^/µL	1.77	1.10–2.86	0.01	1.86	1.11–3.10	0.01	1.86	1.11–3.12	0.01

Data are presented as odds ratios (OR) and 95% confidence intervals (CI) using multiple logistic regression analysis.

Multiple logistic regression model was additionally adjusted with age, sex, tooth brushing frequency, alcohol intake and tobacco smoking in Model 1; BMI was further adjusted in Model 2.

*LDL-C* low density lipoprotein cholesterol, *HDL-C* high density lipoprotein cholesterol.

### Associations of the severer periodontitis and systemic inflammation status for increased cIMT

The moderation effects of systemic inflammation on the association of severer periodontitis for increased cIMT are shown in Table 4. In the univariate analysis, the association for cIMT ≥ 0.8 mm was significant and most remarkably in those with severer periodontitis( +)/higher leukocyte counts( +) [OR: 7.83 (2.89, 21.2)] and then in those with severer periodontitis( +)/higher leukocyte counts(−) [OR: 1.96 (1.09, 3.50)], while compared to those with severer periodontitis(-)/higher leukocyte counts(−). However, the association for cIMT ≥ 0.8 mm in those with severer periodontitis( +)/higher leukocyte counts(−) reduces with adjustments for covariates in Model 1 and Model 2 (ORs 1.92 (1.05, 3.50) and 1.82 (0.98, 3.36), respectively), where the association was not significant in Model 2, indicating the moderation effect of systemic inflammation.

**Table 4:** Multiple logistic regression analysis of severer periodontitis severity and higher leukocyte counts with carotid intima-media thickness ≥ 0.8 mm in military young adults.

	Crude model			Model 1			Model 2		
	OR	95% CI	*p* value	OR	95% CI	*p* value	OR	95% CI	*p* value
Severer periodontitis (−)/higher leukocyte counts (−)	1.00			1.00			1.00		
Severer periodontitis (−)/higher leukocyte counts ( +)	1.55	0.88–2.71	0.12	1.61	0.91–2.85	0.10	1.59	0.90–2.82	0.10
Severer periodontitis ( +)/higher leukocyte counts (−)	1.96	1.09–3.50	0.02	1.92	1.05–3.50	0.03	1.82	0.98–3.36	0.06
Severer periodontitis ( +)/higher leukocyte counts ( +)	7.83	2.89–21.19	< 0.001	7.33	2.62–20.50	< 0.001	6.45	2.21–18.84	0.001

Data are presented as odds ratios (OR) and 95% confidence intervals (CI) using multiple logistic regression analysis.

Multiple logistic regression model was additionally adjusted with age, sex, tooth brushing frequency, alcohol intake and tobacco smoking in Model 1; BMI was further adjusted in Model 2.

Severer periodontitis is defined as periodontitis stage II or stage III and higher leucocyte counts is defined as total leucocyte counts ≥ 7.6 10^3^/µL.

## Discussion

The principal findings of this study were that among young adults who have a low vascular risk burden, periodontitis severity was positively associated with cIMT levels. In addition, there was a dose–response association for clinically increased cIMT with greater periodontitis stages. Systemic inflammation rather than metabolic risk factors and blood pressure was found to be associated with clinically increased cIMT, and systemic inflammation might be a moderator between periodontitis and subclinical atherosclerosis during young ages.

Epidemiological evidence suggested that periodontitis may be associated with a greater risk of incident cardiovascular diseases in adults, and the association was found stronger in younger adults than in older ones^28^, indicating that age was a moderator for the association between periodontitis and atherosclerotic cardiovascular diseases. In addition, the severity of periodontitis may increase risk of atherosclerosis in some animal studies^29,30^. With regard to cIMT, Cairo et al*.* also revealed that severe periodontitis was associated with cIMT ≥ 0.82 mm in young healthy adults, which was in line with the present study finding^31^. On the contrary, López-Jornet et al*.* revealed that periodontitis was associated with carotid atheroma plaques while not cIMT in middle- or old-aged individuals^11^. Range et al*.* also found periodontal pathogens and neutrophil activation within atherosclerotic carotid plaques, supporting a link between severe periodontitis and carotid atheroma plaques formation in older individuals^32^. This study was the first report using physically fit young adults to maximally reduce the effect of age and clarify the association between periodontitis and cIMT.

Periodontitis was considered as a promoter of many systemic diseases, but the signaling pathways for the interconnection remain elusive^3^. In previous studies, various mechanisms linking periodontal disease to cardiovascular disease, e.g., increased cytokines due to inflammation, bloodstream infection, dental plaque accumulation and alveolar bone loss have been proposed in middle- and old- aged individuals^33–37^. In this study, the mechanisms for the association between periodontitis and subclinical atherosclerosis among young adults were mainly reasoned by systemic inflammation assessed by elevated total leukocyte counts. In patients with periodontitis, cytotoxic neutrophil proteases and histones are responsible to ulcer formations and discontinuity on the sulcular epithelium^33^. Daily oral hygiene practices, e.g., tooth brushing and flossing might access the pathogens and its lipopolysaccharide from periodontal pocket to the peripheral blood via the sulcular epithelium, which foster bacteremia and endoxemia^34^. In addition, chronic infection status, e.g., low-grade endoxemia and inflammatory cascade, in which the circulating cytokines bind to the signaling receptors and ligands to generate an inflammatory circle^35^.

Metabolic disorders were common non-communicable diseases and previous studies have shown a bidirectional adverse association with periodontitis^38^. In addition, a large body of evidence from epidemiologic studies also supported an association between metabolic disorders and atherosclerosis^9^. Periodontitis contributes to the progress of atherosclerosis, through the subgingival biofilm, either directly modifying lipid structure by bacteria or indirectly stimulation of inflammatory pathways to affect lipid synthesis and atheroma development^34^. Ylo¨stalo et al. reported an association between periodontal infection and cIMT in middle-aged adults with a low HDL-C level^39^. Metabolic disorders, however, the present study did not show any association with cIMT, possibly because of merely young fit adults included. Age might play a crucial role in this relevance. Atherosclerosis is a process involving inflammation and lipids interaction that is required decades of time to develop clinically significant diseases. In contrast, this study showed periodontitis and inflammatory markers were independent risk factors of clinically greater cIMT, indicating inflammation status is critical for the development of atherosclerosis in young adults.

Although oral hygiene was emphasized and obliged in military in Taiwan that all our study participants were found at least once daily tooth brush, some oral unhealth behaviors, e.g., smoking and alcohol intake were prevalent and might increase the risk of periodontitis. In addition, the oral hygiene status in military was found to be associated with rank^40^ which is highly related to age of military personnel. In this regard, the effects of oral health behaviors on the association between periodontitis and cIMT in military require further investigation.

Some limitations should be considered while interpreting the study findings. First, evidence of the causality of periodontitis for atherosclerosis could not be obtained for the cross-sectional design. Besides, although leucocyte count was a reliable biomarker of inflammation, other inflammatory mediators such as C-reactive protein and interleukin-6 were not available in this study. Moreover, the findings in the military cohort in the present study might not be applicable to the general population of young adults. Finally, more than 90% of the study subjects were men, and thus whether the results were reliable for women may require further study. However, several advantages were present in this study. First, military subjects were characterized by highly homogeneity with unified life style and no regional differences related to access to the dental care, which could reduce unmeasured confounders. Second, there were no participants with diabetes, which has been recognized as an important risk factor for both periodontitis and atherosclerosis.

## Conclusion

This study suggests that periodontitis and increased leukocyte counts within normal limits are independent risk factors of clinically increased cIMT, whereas no associations were present between other metabolic risk factors and clinically increased cIMT in young adults, highlighting the critical role of inflammation status in subclinical atherosclerosis. In clinical practice, an oral examination for the presence of severer periodontitis incorporating a blood test for the presence of increased leukocyte counts within normal limits could be treated as the signs of increased cIMT in young adults which an early prevention of cardiovascular diseases could be taken by physicians. Future study should be aimed to lower the inflammatory status, i.e., periodontitis therapy, in young adults to prevent the following clinical atherosclerotic cardiovascular diseases development.

## Supplementary Information

Supplementary Information.

## Data Availability

The datasets used and/or analyzed during the current study available from the corresponding author on reasonable request.

## Abbreviations

CHIEF: The Cardiorespiratory Fitness and Health in Eastern Armed Forces study
cIMT: Carotid intima-media thickness
