# Supplementary material for: Localized periodontitis severity associated with carotid intima-media thickness in young adults: CHIEF atherosclerosis study
Source: Sci Rep. 2023 Jun 29;13:10523. doi: 10.1038/s41598-023-37840-4 (PMC10310726; doi:10.1038/s41598-023-37840-4)
Supplement: Supplementary file 1 — Supplementary Information. [file 41598_2023_37840_MOESM1_ESM.docx]

**Supplemental Table 1. Clinical Characteristics of Military Young Male Adults Receiving Oral Examination and Carotid Ultrasonography**

|  | Periodontal healthy  (N =319) |  | Periodontitis  (N =133) | | |  | p value |
| --- | --- | --- | --- | --- | --- | --- | --- |
|  |  |  | Stage I  (N =38) | Stage II  (N =56) | Stage III  (N =39) |  |  |
| cIMT, mm | 0.65 ± 0.12 |  | 0.72 ± 0.08 | 0.74 ± 0.05 | 0.76 ± 0.17 |  | <0.001 |
| Age, years | 30.88 ± 5.17 |  | 36.58 ± 3.85 | 29.63 ± 5.56 | 34.18 ± 4.37 |  | <0.001 |
| Alcohol drinking, % | 139 (43.6) |  | 18 (47.4) | 23 (41.1) | 25 (64.1) |  | 0.09 |
| Tobacco smoking, % | 113 (35.4) |  | 15 (39.5) | 21 (37.5) | 20 (51.3) |  | 0.28 |
| Body mass index, kg/m^2^ | 24.85 ± 2.93 |  | 26.60 ± 3.69 | 26.57 ± 3.99 | 28.17 ± 3.69 |  | <0.001 |
| Waist circumference, cm | 83.95 ± 7.29 |  | 89.13 ± 8.88 | 86.62 ± 10.66 | 92.48 ± 9.26 |  | <0.001 |
| Systolic blood pressure, mmHg | 120.37 ± 12.89 |  | 120.05 ± 12.71 | 125.21 ± 18.00 | 124.87 ± 12.78 |  | 0.02 |
| Diastolic blood pressure, mmHg | 72.30 ± 10.40 |  | 75.67 ± 8.32 | 75.57 ± 14.70 | 77.33 ± 10.84 |  | 0.007 |
| Blood examinations |  |  |  |  |  |  |  |
| Total cholesterol, mg/dL | 170.75 ± 29.18 |  | 189.71 ± 28.65 | 190.63 ± 43.76 | 196.44 ± 41.13 |  | <0.001 |
| LDL-C, mg/dL | 103.84 ± 25.00 |  | 121.05 ± 23.99 | 114.57 ± 36.08 | 115.41 ± 32.50 |  | <0.001 |
| HDL-C, mg/dL | 47.97 ± 9.71 |  | 47.37 ± 7.86 | 49.00 ± 9.92 | 48.41 ± 9.56 |  | 0.84 |
| Triglycerides, mg/dL | 107.55 ± 67.55 |  | 148.13 ± 82.38 | 153.46 ± 141.00 | 181.18 ± 163.88 |  | <0.001 |
| Fasting glucose, mg/dL | 92.94 ± 8.21 |  | 104.32 ± 12.41 | 95.18 ± 37.39 | 104.23 ± 14.97 |  | <0.001 |
| Uric acid, mg/dL | 6.82 ± 1.32 |  | 6.80 ± 1.63 | 6.25 ± 1.27 | 6.40 ± 1.51 |  | 0.01 |
| Leucocyte counts, 10^3^/uL | 6.70 ± 1.70 |  | 6.81 ± 1.29 | 6.29 ± 2.09 | 6.58 ± 1.42 |  | 0.36 |
| Tooth brushing frequency |  |  |  |  |  |  |  |
| 1 time/day | 80 (25.1) |  | 4 (10.5) | 7 (12.5) | 7 (17.9) |  | 0.11 |
| 2 times/day | 141 (44.2) |  | 21 (55.3) | 26 (46.4) | 22 (56.4) |  |  |
| ≥3 times/day | 98 (30.7) |  | 13 (34.2) | 23 (41.1) | 10 (25.6) |  |  |

Continuous variables are expressed as mean ± SD (standard deviation), and categorical variables as N (%)

Abbreviations: cIMT, carotid intima-media thickness; LDL-C, low density lipoprotein cholesterol; HDL-C, high density lipoprotein cholesterol

**Supplemental Table 2. Multiple Logistic Regression Analysis between Periodontitis Severity and Carotid Intima-Media Thickness ≥0.8 mm in Military Young Male Adults**

|  | Crude model | | |  | Model 1 | | |  | Model 2 | | |
| --- | --- | --- | --- | --- | --- | --- | --- | --- | --- | --- | --- |
|  | OR | 95% CI | p value |  | OR | 95% CI | p value |  | OR | 95% CI | p value |
| Periodontitis |  |  |  |  |  |  |  |  |  |  |  |
| Stage I | 1.91 | 0.90 – 4.08 | 0.09 |  | 1.64 | 0.68 – 3.91 | 0.26 |  | 1.62 | 0.68 – 3.89 | 0.27 |
| Stage II | 1.88 | 0.98 – 3.59 | 0.06 |  | 1.49 | 0.73 – 3.04 | 0.27 |  | 1.50 | 0.73 – 3.07 | 0.27 |
| Stage III | 3.63 | 1.81 – 7.28 | <0.001 |  | 3.14 | 1.40 – 7.04 | 0.006 |  | 3.17 | 1.41 – 7.12 | 0.005 |
| p value for trend |  |  | <0.001 |  |  |  | 0.007 |  |  |  | 0.007 |
| Tooth brushing frequency ≥3 times/day | 1.01 | 0.63 – 1.62 | 0.97 |  | 1.23 | 0.59 – 2.53 | 0.58 |  | 1.23 | 0.60 – 2.55 | 0.57 |
| Central obesity (waist circumference ≥90 cm) | 2.09 | 1.32 – 3.30 | 0.002 |  | 1.34 | 0.78 – 2.31 | 0.29 |  | 1.45 | 0.70 – 3.00 | 0.31 |
| Hypertension (blood pressure ≥130/85 mmHg) | 1.34 | 0.84 – 2.14 | 0.22 |  | 1.21 | 0.72 – 2.01 | 0.47 |  | 1.22 | 0.73 – 2.05 | 0.45 |
| Total cholesterol ≥200 mg/dL | 2.28 | 1.42 – 3.67 | 0.001 |  | 1.54 | 0.82 – 2.88 | 0.17 |  | 1.54 | 0.82 – 2.88 | 0.17 |
| LDL-C >100 mg/dL | 1.73 | 1.09 – 2.75 | 0.02 |  | 1.32 | 0.75 – 2.32 | 0.34 |  | 1.33 | 0.75 – 2.36 | 0.32 |
| HDL-C <40 mg/dL | 1.03 | 0.58 – 1.83 | 0.92 |  | 0.98 | 0.52 – 1.84 | 0.94 |  | 0.98 | 0.52 – 1.85 | 0.95 |
| Triglycerides ≥150 mg/dL | 1.65 | 1.01 – 2.70 | 0.04 |  | 1.03 | 0.57 – 1.87 | 0.91 |  | 1.05 | 0.57 – 1.92 | 0.87 |
| Fasting glucose ≥100 mg/dL | 1.11 | 0.67 – 1.84 | 0.68 |  | 0.71 | 0.39 – 1.30 | 0.27 |  | 0.72 | 0.39 – 1.31 | 0.27 |
| Hyperuricemia (uric acid ≥7.0 mg/dL) | 1.14 | 0.73 – 1.79 | 0.56 |  | 1.14 | 0.71 – 1.86 | 0.58 |  | 1.15 | 0.71 – 1.86 | 0.57 |
| Leucocyte counts ≥7.6 10^3^/uL | 1.63 | 0.99 – 2.65 | 0.051 |  | 1.74 | 1.03 – 2.94 | 0.04 |  | 1.76 | 1.03 – 2.99 | 0.03 |

Data are presented as odds ratios (OR) and 95% confidence intervals (CI) using multiple logistic regression analysis

Multiple logistic regression model was additionally adjusted with age, teeth brush frequency, alcohol intake and tobacco smoking in Model 1; BMI was further adjusted in Model 2

Abbreviations: LDL-C, low density lipoprotein cholesterol; HDL-C, high density lipoprotein cholesterol
